# Na^+^,K^+^-ATPase as the Target Enzyme for Organic and Inorganic Compounds

**DOI:** 10.3390/s8128321

**Published:** 2008-12-15

**Authors:** Vesna Vasić, Tatjana Momić, Marijana Petković, Danijela Krstić

**Affiliations:** 1 Vinča Institute of Nuclear Sciences, Department of Physical Chemistry, 11001 Belgrade, Republic of Serbia; E-Mails: momict@vinca.rs. (T. M.); marijanapetkovic@vinca.rs (M. P.); 2 Institute of Medicinal Chemistry, University School of Medicine, University of Belgrade, Višegradska 12, Belgrade, Republic of Serbia; E-Mail: krsticdana@yahoo.com (D. K.)

**Keywords:** Na^+^, K^+^-ATPase, toxic agents, inhibition, detection, sensor

## Abstract

This paper gives an overview of the literature data concerning specific and non specific inhibitors of Na^+^,K^+^-ATPase receptor. The immobilization approaches developed to improve the rather low time and temperature stability of Na^+^,K^+^-ATPase, as well to preserve the enzyme properties were overviewed. The functional immobilization of Na^+^,K^+^-ATPase receptor as the target, with preservation of the full functional protein activity and access of various substances to an optimum number of binding sites under controlled conditions in the combination with high sensitive technology for the detection of enzyme activity is the basis for application of this enzyme in medical, pharmaceutical and environmental research.

## 1. Introduction

Molecular interactions on biomembranes play a prominent role in the communication between cells and in signal transduction pathways [1]. Membrane receptors serve as the main targets [2] able to recognize specific ligands selectively, which can trigger a cascade of functional cell responses [3-6]. Because of their regulatory mechanisms and their relevance in ligand - target interactions, membrane receptors are, currently, the focus of detailed biophysical and biochemical investigations directed to elucidate the relation between ligand binding and functional properties, or to resolve structure -activity relationships [7].

Biological membranes are the first fence that has to be overcome by toxic compounds targeting the cell. One of the most important membrane proteins is adenosinetriphosphathase (ATPase, EC 3.6.1.3), an integral part of a sodium-potassium pump and the largest protein complex member of P -type family of active cation transport proteins [8]. It is responsible for establishing and maintaining the electrochemical gradient in animal cells [9-11], due to the free energy resulting from the hydrolysis of an intracellular adenosinetriphosphate (ATP). The sodium pump contributes substantially to the maintenance of the ion concentration gradient throughout the membrane, and enables the animal cell to control its volume and actively transport carbohydrates as well as amino acids. It is also required for nerve and muscle excitation [8].

The minimum functional unit of Na^+^,K^+^-ATPase is an oligomer composed of stoichiometric amounts of two major polypeptides, the so-called α - and β -subunits. The α-subunit, responsible for the catalytic and transport properties of the enzyme, is a multispanning membrane protein with a molecular mass of ~112,000 Da that contains the binding sites for the cations and ATP and acts as the receptor for specific inhibitors, cardiac glycosides such as ouabain, which are bound to the extracellular side of the protein at very high affinity and lead to the inhibition of enzymatic activity [12-14]. The β-subunit is a polypeptide that crosses the membrane once and has a molecular weight between 40,000 and 60,000 Da, depending on the degree of glycosylation in different tissues. The β-subunit is essential for the normal activity of the enzyme [15], and it appears to be involved in the occlusion of K^+^ and the modulation of the K^+^ and Na^+^ affinity of the enzyme [16]. Moreover, there is a third γ subunit, a membrane protein with a molecular mass of 12-14 kDa that interacts only with Na^+^,K^+^-ATPase, thus modulating the enzyme transport activity [17,18]. The γ subunit only interacts with the α – β complex assembled and with functional ability, but not with separate α or β subunits [18]. Na^+^,K^+^-ATPase acts as a dimer (α β-α β). The most widely accepted view related to such a dimmer to act is "flip-flop" model, in which both α subunits show complementary conformations [19]:

$${\text{E}}_{1}{\text{E}}_{2}⇌{\text{E}}_{2}{\text{E}}_{1}$$

where E is the conformation of each α subunit. The observed co-operation between the members of the dimmer [20] supports this model, which postulates that, when one of the α subunits is in the E_1_ conformation, the other one is necessarily in the E_2_. Biochemical and spectroscopic data show that long-range E_1_-E_2_ conformational transitions in the α-subunit mediate interactions between cytoplasmic domains and the cation sites in the intramembrane domain [9, 21, 22]. These transitions couple the scalar processes of ATP binding, phosphorylation, and dephosphorylation to the vectorial extrusion of three Na^+^ ions and uptake of two K^+^ ions.

The sodium pump is characterized by a complex molecular heterogeneity that results from the expression and differential association of multiple isoforms of both its α- and β-subunits. Individual genes of four α (α_1_, α_2_, α_3_ and α_4_)-subunit isoforms and at least three β (β_1_, β_2_ and β_3_) -subunit isoforms of Na^+^,K^+^-ATPase have been identified in mammalian cells [23-25]. The different kinetic parameters for activating cations (Na^+^ and K^+^), the substrate ATP, and ouabain, for each Na^+^,K^+^-ATPase isozyme imply that each isoform has distinct properties [26-28]. The distribution of the Na^+^,K^+^-ATPase α and β subunits isoforms is tissue- and developmental-specific, suggesting that they may play specific roles, either during development or coupled to specific physiological processes [25, 29, 30]. The α_1_ isoform is ubiquitous and it is the major isoform in the kidney and many other tissues, while the α_2_ isoform is the predominant one in skeletal muscle. All three isoforms are found in the brain, the α_3_ isoform is located essentially in neurons, while the α_2_ isoform is found in astrocytes and some limited neuronal populations. Interestingly, the α_4_ isoform is found exclusively in the mid region of the sperm tail [31]. However, the α_1_ and α_2_ isoforms carry out different physiological roles. The α_2_ isoform appears to be involved in regulating Ca^2+^ transients involved in muscle contraction, while the α_1_ isoform probably plays a more generalized role. It is thus possible that the α_2_, α_3_, and α_4_ isoforms are involved in specialized functions of various tissues, helping to explain their tissue- and developmental-specific regulation [25, 29-31].

The expression of Na^+^,K^+^-ATPase isoforms can be altered by pathological conditions. For instance, in several cardiac diseases, the Na^+^,K^+^-ATPase isoform composition of the heart is modified [32]. Numerous studies have reported changes in Na^+^,K^+^-ATPase subunit expression and activity in the course of malignant transformation [33-36], including gliomas [37], with evidence that these occur at the very early stages of tumorigenesis [35]. Moreover, it was previously shown that both non-small cell lung and prostate cancer overexpress Na^+^,K^+^-ATPase α_1_ compared with healthy tissues [35, 36, 38], while reduced expression of the β_1_ isoform is commonly observed in carcinoma and is associated with events involved in cancer progression [39]. Recent studies show that in addition to pumping ions, Na^+^,K^+^-ATPase interacts with neighboring membrane proteins and organized cytosolic cascades of signaling proteins to send messages to the intracellular organelles [40, 41]. Moreover, it seems that two pools of sodium pumps exist, *i.e.*, one broadly distributed in the plasma membrane directly involved in ion exchange, and another (the one located in caveolae) implicated in the signal transduction in couple with tyrosine kinase Src and epidermal growth factor receptor and functions as a signal-transducing receptor for cardiotonic steroids [36, 38, 40, 41]. These data support the view that Na^+^,K^+^-ATPase could be an important target for the development of anti-cancer drugs as it serves as a versatile signal transducer, it is a key player in cell adhesion and the aberrant expression of Na^+^,K^+^-ATPase and activity are implicated in the development and progression of different cancers [35-38].

The sodium pump is specifically inhibited by a series of naturally occurring cardiac glycosides, a family of compounds that includes cardenolides and cardiotonic steroids. The ouabain like specific inhibitors of sodium pump activity primarily bind to extracellular domains of α subunits [42]. Since cardiotonic steroids are the natural ligands and specific inhibitors of the sodium pump [43], this supports the possibility of their development as anticancer agents targeting overexpressed Na^+^,K^+^-ATPase α subunits [35-38, 44].

However, the activity of Na^+^,K^+^-ATPase may be affected by various endogenous and exogenous factors [42-54]. The regulation of Na^+^,K^+^-ATPase activity in various tissues is under the control of a number of circulating hormones that impart both short- and long-term control over its activity [44, 45]. Also, the activity of the enzyme is dependent on the lipid status of the membrane [46]. Although the mechanism of the toxic effect of various Na^+^,K^+^-ATPase activity modulators has not been completely understood yet, this enzyme can be taken as meaningful index of cellular activity and represents a useful toxicological tool [54].

Considering the key role of sodium pump in normal functioning of most cells of higher eukaryotic organisms, this review is an attempt to summarize the information regarding the alterations of Na^+^,K^+^-ATPase activity by various specific and non specific modulators, as well as the basis for application of this enzyme as bioanalytical tool in medical, pharmaceutical and environmental research.

## 2. Inhibition of Na^+,^K^+^-ATPase activity

There are great number of different drugs and environmental toxicants which modulate the sodium pump activity in the concentration dependent manner [42-54]. The most frequently investigated sodium pump inhibitors are natural and synthesized cardiotonic steroids and their derivatives [55], endogenous ouabain like inhibitors, isolates from natural plants, pharmaceutically important organic and inorganic compounds, antitumor drugs, biologically relevant metal ions, pesticides, herbicides and organic solvents. Table 1 gives the overview of some of the most potent sodium pump inhibitors from the group of ouabain like drugs, platinum group anticancer drugs, biologically relevant and heavy metal ions and agents used as herbicides and insecticides. These inhibitors will be discussed in more details in this chapter.

Numerous *in vitro* and *in vivo* methods are usually used to assess the inhibition of Na^+^,K^+^-ATPase activity in inhibition studies, as well as the enzyme preparation from different animal or human tissues [44-53]. Measurement of the specific Na^+^,K^+^-ATPase activity (nanomol of inorganic phosphate, Pi, liberated per min per milligram of protein as well as protein content) is required to evaluate the purity or to determine the actual specific enzyme activity [56-58]. Enzymatic ATP hydrolysis has been measured by several different means, as well as the determination of adenosine diphosphate (ADP) by enzyme coupling [59], or by colorimetric reactions [60, 61] and spectroscopic techniques [62], including measurement of ^32^P release from [γ-^32^P]ATP hydrolysis [63, 64]. In general, non-radioactive assays are much less sensitive than the radioactive ones. The RIA (radioactive immunoassay) method based on ^32^P is technically simple enough to enable simultaneous measurement of the enzyme activity in large number of tubules and sufficiently sensitive to determine enzyme activity in each region of the nephron [59, 63]. However, in some cases the easiest, quickest and most sensitive is the spectrophotometric assay, based on the conversion of the released orthophosphate into molybdato-phosphoric acid and its extraction with organic solvent [65]. This is particularly important for enzyme kinetics studies, were nano molar concentrations of ATP are needed.

There are also some commercially available ATPase colorimetric assay kits [66]. These kits contain specially purified Pi-free ATP to ensure the lowest possible background signals. They also contain the additives to prevent background signals arising out of nonenzymatic ATP hydrolysis. Assays can be read anywhere in the wavelength range 590-660nm.

The Na^+^,K^+^-ATPase used in the inhibition studies could be isolated from different sources (rat brain, dog kidney, porcine cerebral cortex, human blood) [48, 52, 54, 67]. Each choice of the Na^+^,K^+^-ATPase source, *i.e.* the choice of animal species or type of tissue, as well as isolate purity (tissue homogenates, cell membranes, commercial enzyme) has its assets and drawbacks. The asset of use of tissue homogenate in inhibition studies is that effects of enzyme activity modulators are the most comparable with modulator effects *in vivo*. Unfortunately, the data concerning molecular mechanism of inhibition could not be obtained by use of tissue homogenate. To test the possibility that some tissue component(s) may protect enzyme, some authors compared the effects of inhibitors in homogenates and microsomes of the same tissues [68]. The IC_50_ values in homogenates were 2–5 times higher than the values in the respective microsomes [68, 69]. Hence, purified enzyme preparation must be used for molecular interaction studies between enzyme and inhibitor (e.g. type of inhibition, IC_50_, kinetic parameters), in order to avoid the false interpretation of the enzyme inhibition.

Also, the choice of Na^+^,K^+^-ATPase source is dependent on the enzyme modulator under the investigation. If the tested modulator exerts the high affinity towards the certain isoenzyme, the tissue with target isoenzyme expressed is usually chosen. Moreover, molecular biology techniques enable cloning of desired isoenzymes (α and β isoforms). The drawback of this approach is the difference in the sensitivity of native and expressed Na^+^,K^+^-ATPase. The rat α1β1 enzyme expressed in insect cells is over 2-fold more sensitive and the α_3_β_1_ approximately 20-fold less sensitive to ouabain than the native enzyme. This difference in sensitivity between the native and expressed Na^+^,K^+^-ATPase may be a result of the different lipid composition environment of the enzyme [25].

### 2.1. Specific inhibition by cardiac glycosides

It is well known that the family of compounds, that includes cardenolides and cardiotonic steroids, such as digitoxin, digoxin, digitoxigenin, medigoxin and some derivatives (acetyldigitoxin, acetyldigoxin, methyldigoxin), are very potent and specific inhibitors of Na^+^,K^+^-ATPase activity in heart, renal and red blood cells [42,70-77]. When used as therapeutics, the concentrations of digitalis compounds produce a moderate enzyme inhibition (about 30%), since the concentrations that reach toxic levels inhibit over 60% of the enzyme activity [74, 78].

**Figure 1.**
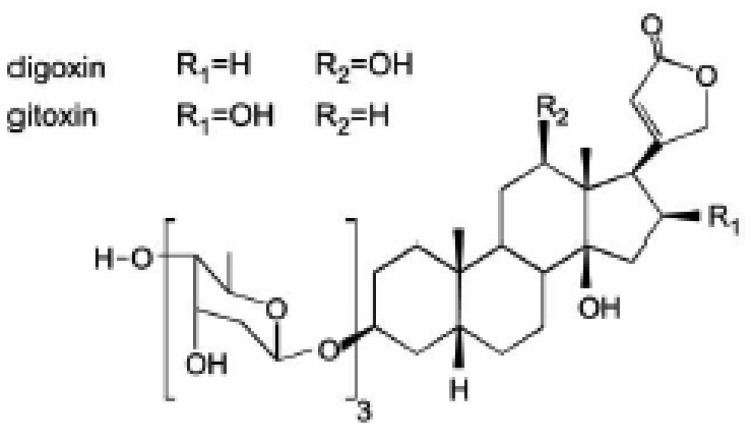
Basic structure of some specific Na^+^,K^+^-ATPase inhibitors.

**Figure 2.**
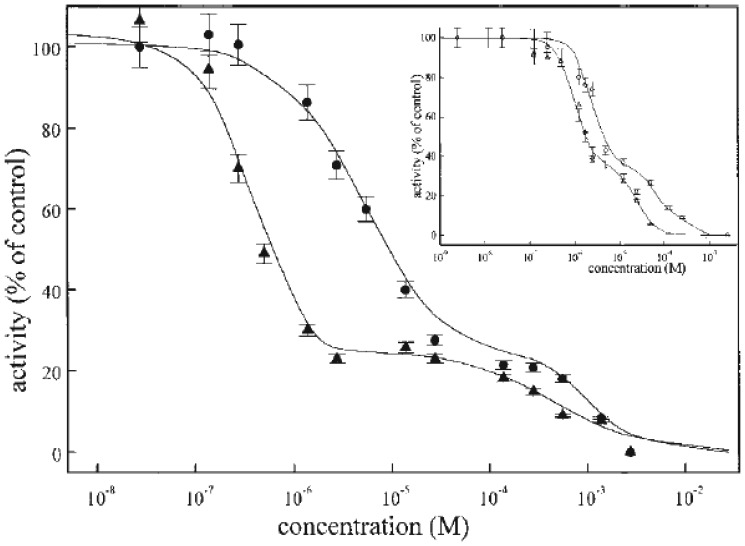
Inhibition of Na^+^,K^+^-ATPase activity by digoxin (circles) and gitoxin (up triangles) in human erythrocyte membrane and commercial porcine cerebral cortex (inset). The solid lines represent the theoretical curves assuming two-site model fit, using experimentally determined IC_50_ values for high and low affinity isoenzymes. Reproduced from [67].

The inhibition of Na^+^,K^+^-ATPase activity induced by two isomers, digoxin and gitoxin (Figure 1), was investigated in our laboratory [67, 75]. It is noteworthy that while digoxin is one of the most frequently used cardiotonic drugs, gitoxin, which has the same chemical formula, but differs in the position of –OH groups, exerts toxic effects. The inhibitory power of these two compounds was tested by their ability to alter the activity of Na^+^,K^+^-ATPase isolated from human blood erythrocyte and from purified commercial synaptosomal porcine cerebral cortex membranes (Figure 2). Red cell membranes were prepared according to the method of Post *et al.* [79] with certain modifications. The both enzyme preparations were assayed in a standard incubation medium [67, 75] in the presence or absence (control) of the desired concentration of inhibitor. The reaction was allowed to precede 10 min for cerebral cortex and 1 h for human erythrocytes, before monitoring the enzyme activity, using standard spectrophotometric assay. The results show, that digoxin and gitoxin inhibited Na^+^,K^+^-ATPase in both preparations in a concentration dependent manner, but with diverse potency [80]. However, the human blood erythrocyte Na^+^,K^+^-ATPase was more sensitive to exposure to gitoxin, compared to porcine cerebral cortex. In addition, the biphasic inhibitory curves were obtained in both enzyme preparations, indicating the interference of two distinct inhibitor binding sites. The heterogeneity of digoxin sites has also been reported in ox and rat brain Na^+^,K^+^-ATPase and related to high and low affinity isoforms of α subunit [81, 82].

**Figure 3.**
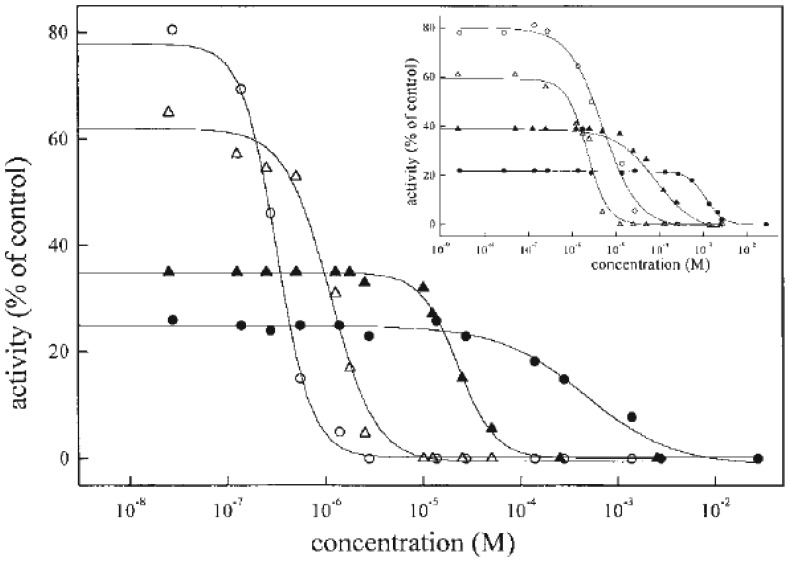
The theoretical curves for inhibition of high (open symbols) and low (solid symbols) affinity Na^+^,K^+^-ATPase isoenzymes induced by gitoxin and digoxin (inset). Circles—human ghosts membranes; up triangles—commercial porcine cerebral cortex. Reproduced from [67].

The measured activity was ascribed to the overall activity of the high and low affinity isoforms. In the mathematical analysis of the results (Figure 2) it was assumed that the mass action principles were fully satisfied [67, 80] and that the plot of the total activity represents the line for “two enzymes acting on one substrate” [83, 84]. The computer program was set up for the analysis of the data, assuming a two-site model fit. In the first approximation the half maximum inhibition concentrations (IC_50_ values) for the high and low inhibitor affinity isoenzymes, respectively, were calculated by fitting the experimental results to the sum of two sigmoid curves. The theoretical curves for high and low affinity enzyme isoenzymes were derived from the approximated IC_50_ values by several iterations, and were derived from the calculated IC_50_ values and presented in Figure 3. It is obvious from the inhibition curves that the same ratio of isoenzymes was obtained in both preparations. The high affinity of porcine cerebral cortex Na^+^,K^+^-ATPase of porcine cerebral cortex Na^+^,K^+^-ATPase of porcine cerebral cortex Na^+^,K^+^-ATPase to digoxin and gitoxin probably can be attributed to isoenzyme(s), containing α3 isoform. It is well known that α3 isoform is especially abundant in the brain and some other vertebrate tissues [55], as well as α_3_ isoforms from rat brain or dog heart are the most sensitive towards the cardiac glycosides [25]. Western analysis of the Na^+^,K^+^-ATPase from mature human erythrocyte ghosts, purified by ouabain column chromatography, has shown that erythrocytes contain the α1 and α3 isoforms of the α subunit [55]. However, as previously shown for human Na^+^,K^+^-ATPase isoenzymes expressed in *Xenopus* oocytes, all human isoenzymes have a similar affinity for ouabain [26]. Finally our results can be explained by isoform-specific differences which exist in K^+^/ouabain antagonism and probably as previously shown, protect α_1_ but not α2 or α3 from digitalis inhibition at physiological K^+^ levels [26]. From this reason, low affinity of Na^+^,K^+^-ATPase towards digoxin and gitoxin can be ascribed to isoenzyme(s) containing α_1_ isoform, which is also present in porcine cerebral cortex and inhibited with similar affinity towards investigated digitalis.

**Figure 4.**
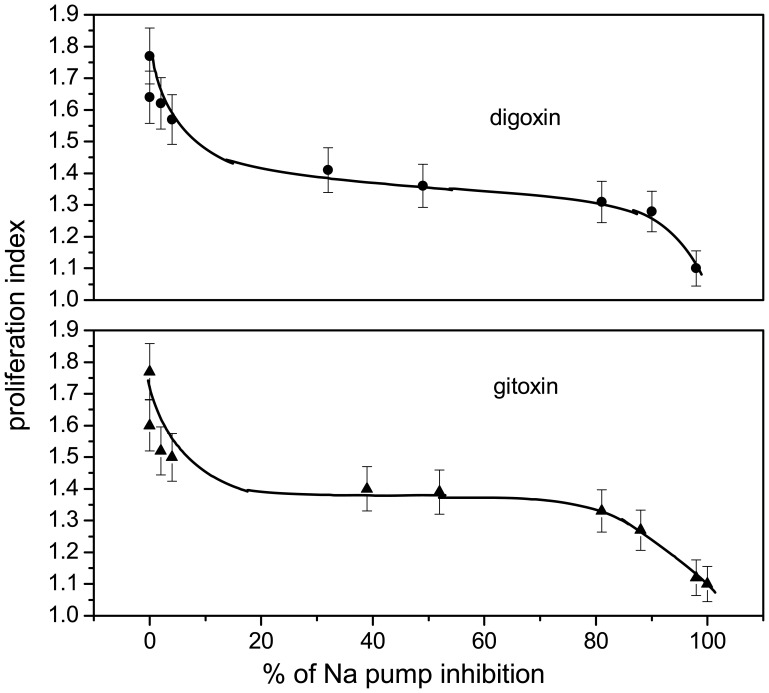
Dependence of proliferation index in harvested human lymphocytes exerted to digoxin and gitoxin on % Na^+^,K^+^-ATPase inhibition

Toxicity studies of these compounds were extended by measuring the proliferation index and micronuclei incidence in the harvested human lymphocytes cell cultures. The correlation between the proliferation index and the activity inhibition (Figure 4) suggested the similar behavior of two isomers – specific inhibitors of Na^+^,K^+^-ATPase. On the contrary, only the increasing concentrations of gitoxin induced the increase of micronuclei frequency in the harvested lymphocytes cell cultures. Briefly, gitoxin concentration increased from 5x10^-9^ – 5x10^-6^ M induced the 3 fold increase of genetic damage in cell cultures. Besides, digoxin did not exert any effects on the micronucleus expression in the same concentration range.

The mechanism of inhibition of high affinity isoenzymes of Na^+^,K^+^-ATPase activity by ouabain like cardiac glycosides undergoes to the Michaelis-Menten kinetics [76, 67, 80-82]. Kinetic analysis of the results showed that digoxin and gitoxin behave as noncompetitive inhibitors of Na^+^,K^+^-ATPase activity, which interfered with the enzyme by binding to the enzyme - substrate complex causing the structural distortion of the active sites [76].

In naturally occurring digitalis glycosides the unsaturated γ- and δ-lactones present at the 17β-position of the steroidal skeleton are associated with high affinity for the Na^+^,K^+^-ATPase receptor [86, 87]. Recently, it was confirmed that basicity, *i.e.* a strong ionic interaction between one of carboxylate residues present in the α-subunit of the Na^+^,K^+^-ATPase and the cationic form of some digitalis like derivatives is relevant for interference with enzyme activity [55, 87]. The presence of -OH groups at different positions of the steroidal skeleton (Figure 1) reduces, in general, the interaction energy, though it depends on the location and spatial disposition of such –OH groups [88, 89].

Also, the inhibition of porcine cerebral cortex Na^+^,K^+^-ATPase by oleandrin and its derivative oleandrigenin, which is structurally similar to digoxin, confirms that they likely exert their toxic effects through inhibition of sodium pump activity [90]. An endogenous digitalis - like factor (EDLF) present in human urine and plasma is an inhibitor of the cell membrane Na^+^,K^+^-ATPase [91]. Its concentration may increase in arterial hypertension, during body volume expansion and in prenatal life [92, 93].

The novel cardenolide UNBS1450, obtained by hemi-synthesis from naturally occurring 2″-oxovoruscharin [94, 95], which is known to bind to the sodium pump and display potent anti-tumor activity both *in vitro* and *in vivo* in experimental models of human cancer, structurally differs significantly from the classic cardiotonic steroids ouabain, digitoxin, and digoxin, but demonstrates much higher rates of inhibition of all Na^+^,K^+^-ATPase isozymes (notably, α1β1) than classic cardenolides. In this study the attempt was made to propose that the sodium pump, and more specifically, its α1subunit, is a new target in the context of malignant glioblastoma (GBM) treatment.

### 2.2. Platinum-anticancer drugs

**Figure 5.**
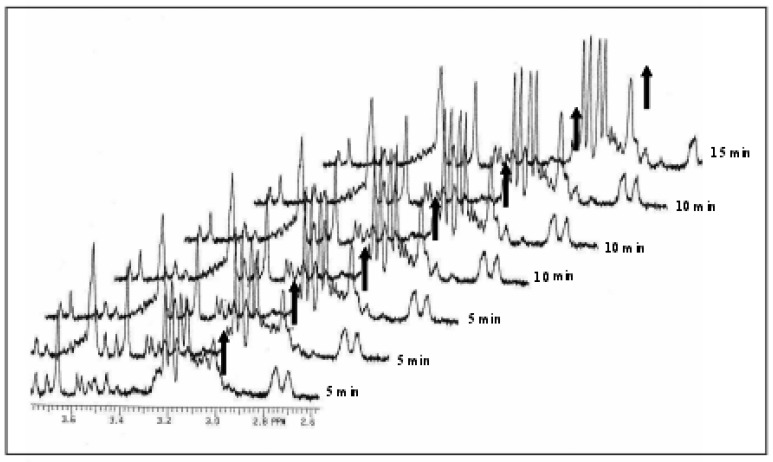
^1^H-NMR spectra of the reactions of [PdCl(dien)]^+^ (5 mM) with enzyme as a function of time. Reproduced from [100].

The complexes of platinum group elements have received much attention over last two decades, because of their potential antitumor activity and increasing applications in chemotherapy [96-99]. For the study of the reaction mechanism of platinum (II) complexes their palladium analogs are often used as model compounds, since they exhibit about 10^4^–10^5^ fold higher reactivity, while their structural and equilibrium behavior is very similar. Recently published results concentrated on the reactions of Pt(II) and Pd(II) complexes with sulphur-bonding ligands, such as l-cysteine and glutathione (GSH), which could be of fundamental importance for understanding the toxicity and interactions with proteins of related platinum complexes [100,101]. These complexes have a great affinity for substitution reactions of the Cl^-^ ligand by SH-donors. The coordinative interaction between [PdCl(dien)]^+^ and enzyme was verified ^1^H NMR spectra (Figure 5), as well as by UV spectrophotometry. Moreover, the similarity in the changes in absorption spectra of [PdCl(dien)]^+^ in the presence of Na^+^,K^+^-ATPase and thiols (L-cysteine and GSH) suggested that the complex ion interacts with enzymatic sulfhydryl groups.

**Figure. 6.**
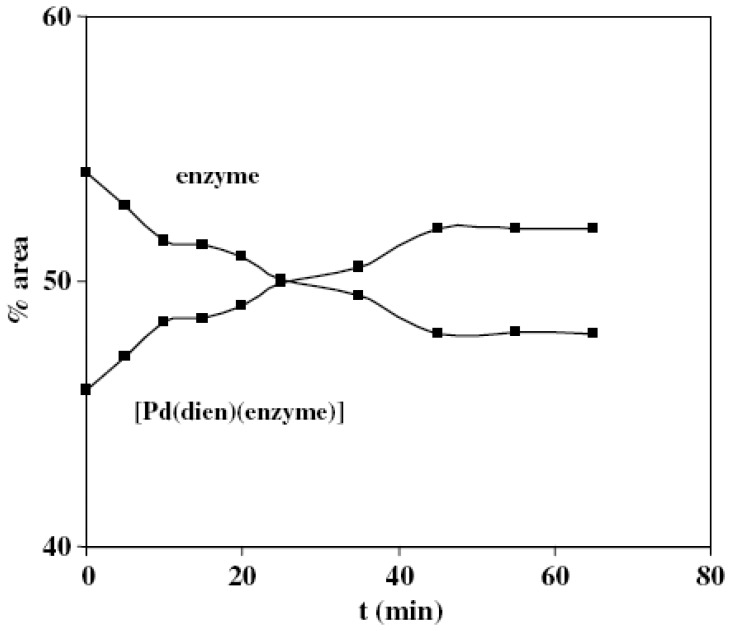
Product formation during the reaction of [PdCl(dien)]^+^ with porcine cerebral cortex Na^+^,K^+^-ATPase. Calculations were performed by relative integration (estimated error 5%) of suitable proton signals of reaction product and starting materials during reaction. Reproduced from [100].

The time course of the enzyme – inhibitor complex formation derived from^1^H NMR data is presented in Figure 6. The kinetic analysis exhibited typical Michelis-Menten noncompetitive type of inhibition. It seems that substrate and inhibitor were bonded to the different sites on enzyme, and the binding of the inhibitor did not affect binding of the substrate. Furthermore, Na^+^,K^+^-ATPase can be observed as SH-donor ligand since the enzyme has 36 SH groups which are held responsible for interactions of this enzyme with various metal ions [102-104].

However, the stability constants of enzyme-Pd (II) complexes determined in our work (Ks=1/Kiࣅ10^4^ M^-1^) were close to the value of overall binding constant that was reported for the interaction of Na^+^,K^+^-ATPase with cisplatin (K=1.93×10^4^ M^-1^) [97, 98], but are also for two orders of magnitude lower compared to aqua complexes of heavy and transition metals [102, 103].

Our results reported that inhibitory effects can be prevented by –SH containing ligands. Moreover, the dose-dependent recovery effect on inhibited Na^+^,K^+^-ATPase activity exposed to Pd(II) complexes was obtained in the presence of l-cysteine and GSH [97-99]. Full recovery was achieved when concentration of SH-containing ligands was equal or higher than the Pd (II) complex concentration. The recovery of the inhibition can be explained by high potency of thiols to extrude Na^+^,K^+^-ATPase from the enzyme-inhibitor complex and formation of stable Pd(II)-thiol complexes [105]. Since the intracellular concentration of GSH is up to 8 mM, and is usually much greater than those of cysteine, the ability of these ligands to detoxify after chemotherapy seems to be interesting for further investigations.

As mentioned above, cisplatin (*cis*-diaminedichloroplatinum) is also a potent anticancer drug [106-108]. It is well known that cisplatin inhibits Na^+^,K^+^-ATPase, but rather a high cisplatin concentration (280-800 μM) and/or a long period of incubation (60-90 min) was required to obtain about 50% inhibition of enzyme activity. The toxicity of the cisplatin has been ascribed to its interactions with thiol groups of the proteins, and the toxic side effects of platinum antitumor complexes are probably the result of inactivation of certain enzymes due to binding to the thiol groups of cystein residues [106]. Spectroscopic evidence of cisplatin-ATPase complexes showed that at low drug concentration (0.1 μM), cisplatin binds mainly to the lipid portion of Na^+^,K^+^-ATPase, through lipid carbonyl group. At higher Pt (II) concentration, drug-enzyme binds extends to polypeptide -C=O and -C–N groups. At high cisplatin concentration (1 mM), drug binding results in protein secondary structural changes, mainly for the α-helix, β-structure and random [107]. The evidence was provided that Na^+^,K^+^-ATPase α1 subunit plays an important role in the transport of cisplatin in cells [108].

Inhibition of Na^+^,K^+^-ATPase by chlorauric acid (Au^3+^) and gold sodium thiomalate (Au^+^) was studied in dog brain and kidney and in human kidney enzyme preparations [68]. The extent of inhibition is similar to the potency of Pt (II) and Pd(II) complexes [100, 101], as well as ouabain and vanadium [81, 82], the classical –SH inhibitors of the enzyme. In fact, the action of gold is more complex than the simple inhibition of –SH groups and can hardly be transpolated in vivo because the reduction of Au^3+^ to Au^+^ can occur and the two oxidation states of gold behave differently toward the enzyme. Au^+^ inhibited Na^+^,K^+^-ATPase 2 to 3 times more effectively than did Au^3+^. The inhibitory action of Au^3+^ (but not Au^+^) was potentiated by ascorbic acid, suggesting reduction of Au^3+^ to Au^+^ by ascorbic acid. Besides, reduced metallic gold particles may be deposited in the kidney with a loss of activity. However, gold is one of the most potent nonspecific inhibitors of Na^+^,K^+^-ATPase, with characteristics differing from other metallic inhibitors of this enzyme system [68].

### 2.3. Metal ions

Hydrolysis of ATP catalyzed by Na^+^,K^+^-ATPase depends sensitively on the presence of metal ions, such as Na^+^, K^+^ and Mg^2+^. However, a great number of biologically important metal ions which are, at trace levels, necessary to support life, at elevated levels become toxic, built up in biological systems, and induce significant health hazards [102-104, 109-112]. Many investigators have studied the *in vitro* effects of exposure to metal ions on synaptosomes, which have more nonspecific metal binding sites than SPMs. For instance, Na,K-ATPase from the kidney contains 36 sulfhydril groups with 34 of them found in the catalytically active α subunit [113, 114].

Recently, we investigated the *in vitro* influence of some metal ions of first transition series elements (Zn^2+^, Fe^2+^, Co^2+^, Cu^2+^) and heavy metals (Co^2+^, Hg^2+^, Pb^2+^) on Na^+^,K^+^-ATPase activity using rat synaptic plasma membranes (SPM) in native and immobilized form as a model system [104, 115]. It can be assumed that induced inhibition depends sensitively on. their affinity to form complexes with the ligands containing –SH, –NH_2_ or –COOH groups, but it is now generally accepted that the most toxic effect of heavy metals is due to their binding to sulfhydril groups of enzymes. It is reasonable to assume that the ion free activation energy (ΔG°′) for water exchange in metal ion aqua complexes play dominant role concerning the potency of their interference to the Na^+^,K^+^-ATPase activity. Therefore, the metal toxicity, expressed as IC_50_, was correlated with the stability constants of corresponding metal – cysteine complex (Table 2) and also with the solubility constants of metal - S^2^-complex of respective metal sulfide. Because of affinity of many metal ions to bind to adenosine nucleotides, the monitoring of the concentration of MgATP^2-^, which is the true substrate for both ATPase activities, and other key species, such as Mg^2+^ and ATP^4-^, as well the free metal ion concentration was also required [103, 112].

Heavy metals, such as Pb^2+^, Cd^2+^ and Hg^2+^ exerted a potent inhibitory effect on Na^+^,K^+^-ATPase isolated from different tissues, like rat brain and rat liver, by binding avidly to sulfhydryl groups with similar affinities [116, 117]. Sensitivity of enzyme toward Cd^2+^ and Hg^2+^ was increased after its immobilization by adsorption on a nitrocellulose membrane. On contrary, the immobilization of enzyme by adsorption on polystyrene microtiter plates had no affect on enzyme sensitivity toward same ions [104]. Inhibitory effects of Cu^2+^, Zn^2+^ and Fe^2+^ on bovine cerebral cortex Na^+^,K^+^-ATPase activity have been obtained and the extent of inhibition seemed too dependent on the presence of chelators [103, 112].

Kinetics analysis showed that the nature of enzyme inhibition is non competitive. The inhibitory effects of by Fe^2+^, Co^2+^, Zn^2+^ and Cu^2+^ can be prevent by addition of strong metal-ion chelators such as EDTA, L-cysteine or glutathione and is dose dependent. Recovery of the Hg^2+^-induced inhibition was not achieved, even when the chelators were present at concentration above 0.01 M [103, 112].

The synergistic effects with binary combinations of heavy metals on the activity of Na^+^,K^+^-ATPase using the mixtures of Cu/Zn, Cu/Fe, Zn/Fe, Pb/Cd, and Cu/Pb/Zn/Cu ions were obtained in all cases. Moreover, all metal ions in the mixture at concentration levels near IC_50_ values inhibited the enzyme activity completely [103]. The inhibition induced by combination of Pb with Cd was time dependent. Addition of 1mM EDTA in the medium assay recovered 100% of the inhibited enzyme activity [118].

### 2.4. Toxic organic compounds

#### 2.4.1. Herbicides

Chlorinated phenols are intermediates in the production of chlorophenoxy herbicides and are also used directly for wood preservation [119]. These compounds inhibited the Na^+^,K^+^-ATPase of Chinese hamster ovary cells. The effects of chlorphenoxyl herbicides and their metabolites on Na^+^,K^+^-ATPase of erytrocyte plasma membrane, isolated from human blood were published. Investigated basic compounds 2,4-dichlorophenoxyacetic acid (2,4-D), 4-chloro-2-methylphenoxyacetic acid (MCPA), and 2,4,5-trichlorophenoxyacetic acid (2,4,5-T) caused an increase in the activity of the enzyme at low concentration (1mM) but a decrease at higher concentration. For the metabolites (2,4-dichlorophenol, 4-chloro-2-methylphenol, 2,4-dimethylphenol, and 2,4,5-trichlorophenol) a decrease in the activity of ATPase was shown [120]. The changes of Na^+^,K^+^-ATPase activity may be a result of changes in lipid composition and fluidity of the erythrocyte membranes, but these claims are controversial. Some authors suggest that a decrease in membrane fluidity (after an increase of cholesterol content) might cause a stimulation of Na^+^,K^+^-ATPase activity in human erythrocyte membrane. Other authors claim the opposite effect. Phenoxyl herbicides, as suggested by some authors may incorporate into cellular membrane, and perturb its structure and function [121], but also to increase the plasma membrane fluidity.

Abnormalities in lipid composition and structure may cause the changes in the Na^+^,K^+^-ATPase activity of cell membranes [122-124]. The activation of Na^+^,K^+^-ATPase also correlates with ability for interactions with various phospholipids and the fluidity of the fatty acyl chains [124] of the surrounding membrane. The membrane-crossover experiments prove that the membrane lipids are important determinants of Na^+^,K^+^-ATPase molecular activity. The inhibition of erythrocyte Na^+^,K^+^-ATPase was associated with membrane protein cross-linking and lipid peroxidation [125,126]. In addition, phenol compounds may induce hydroxyl radical, which might damage protein structure [127]. 2,4-D-induced decrease in the Na^+^,K^+^-ATPase activity was observed in analysis of the effects of the herbicide on human skin fibroblast [128]. A decrease in Na^+^,K^+^-ATPase activity was reported in vesicles of rabbit brain choroids plexus [129]. The increase in free –SH group content may imply that phenoxyl herbicides damage the enzyme molecule structure by disruption of disulfide bridges [130]. These disruptions lead to enzyme inactivation. Both mechanisms, changes in the cellular membrane or protein damage lead to or result from protein conformational changes and thus, exhibit as enzyme activity changes.

#### 2.4.2. Insecticides

It is known that ATPases are target enzyme for organochlorine pesticides, which affect conduction of nerve impulses [131]. The chlorinated hydrocarbon insecticide chlordecone inhibits Na,K-ATPase [132]. Dieldrin and methyl mercury are inhibitors of rat brain synaptosomes Na^+^,K^+^-ATPase [133, 134]. Methyl mercury binds to sulfhydryl containing proteins [135].

Some organophosphorus compounds (ethion, chlorpyrifos, dimethoate and monocrotophos) are reported to inhibit the activity of erythrocyte and rats SPM Na^+^,K ^+^-ATPase[136-139]. Parathion inhibited renal [140] and pig kidney Na^+^,K^+^-ATPase [141], since methyl parathion inhibited rat synoptosomal Na^+^,K^+^-ATPase [69]. An indirect mechanism of the interaction was proposed: these organophosphorous insecticides could inhibit the activity of the Na^+^,K^+^-ATPase by excluding the enzyme protein from its normal lipid milieu [141]. Malathion was also reported as Na^+^,K^+^-ATPase inhibitor [142]. Synthesized phosphorotionate 2-butenoic acid-3-(diethoxyphosphinothioyl)-methyl ester (RPR-II) inhibited rat brain Na^+^,K^+^-ATPase [143].

Inhibition of Na^+^,K^+^-ATPase by an insecticide may be due to its binding to the ATPase molecule at or near the ouabain site [144] or may be a result of its interaction with the dephosphorylation step of the phosphoryl intermediate of the enzyme [145]. Alternate modes of interaction are also possible [136]. In the case of some chlorinated hydrocarbon insecticides (chlordecone), the inhibition of Na^+^,K^+^-ATPase in the central nervous system may be casually related to their neurotoxicity [146].

## 3. Principle of the application of Na^+,^K^+^-ATPase as a bioanalytical tool

Since cellular membranes are among the first targets for therapeutic or toxic activity in pharmaceutical, medical and environmental research, Na^+^,K^+^-ATPase is an excellent model system for elucidation of these effects induced by different drugs and environmental toxicants. High sensitivity of this enzyme to broad spectrum of different agents makes it suitable for toxicological and environmental analysis, but the lack of selectivity makes it unsuitable for a specific sensor. However, several efforts to develop the Na^+^,K^+^-ATPase based biosensor or high-throughput cell-based functional assay for Na^+^,K^+^-ATPase, discussed in the present and next chapters, are based on enzyme immobilization and choice the appropriate method for enzyme activity measurements.

Enzymes immobilized on porous or non-porous supports can be reused and show better thermal and chemical stability [147-150]. This is the general concept for development of biosensors. In addition, immobilized enzyme is easily separated from the test solution and its loss is prevented by immobilization. A literature survey offers several methods, such as *i)* physical adsorption, *ii)* covalent bonding, *iii)* cross-linking, *iv)* inclusion and *v)* encapsulation for the immobilization of enzymes [151].

Different supports can be used for enzyme immobilization. They can be separated according to their chemical nature (e.g. metal colloid particles, metal oxides, synthetic or natural polymers, etc.), structure (thin films, particles, spheres, etc.) or dimensions of their diameters (micro-, nanometer). The choice of a support is in close relationship with the main purpose of immobilization.

An immobilization technique (approach) must be carefully selected for each enzyme-support combination in order to preserve the enzyme properties such as structure and, consequently, enzyme activity and selectivity. In addition, the nature of the bond between enzyme and support has to be sufficiently strong to avoid lost of an enzyme after the assay and to enable multiple applications of the same enzyme preparation. Simultaneously, this bond has to be sufficiently flexible to enable the enzyme-substrate interaction and the enzyme activity. Once such a method has been developed, it can be stated that the particular enzyme is functionally immobilized.

The main problem in all toxicological study involving Na^+^,K^+^-ATPase, particularly enzyme present in the synaptosomal plasma membranes, is the rather low time and temperature stability of the SPM enzyme preparations. Therefore, in order to fully utilize the sensitivity of this enzyme towards a broad spectrum of compounds, as discussed in previous section, it is necessary to improve its stability by immobilization.

Although there is a lack of the literature data about Na^+^,K^+^-ATPase immobilization, a few publications concerning this problem have appeared in the last decade [104]. Procedures for immobilization of Na^+^,K^+^-ATPase, or more precisely, of Na^+^,K^+^-ATPase-enriched membrane fragments, are mostly based on the simple physical adsorption of membrane fragments to the surface of support, but there is also an example on the covalent cross-linking of Na^+^,K^+^-ATPase and binding to dextran surface.

### 3.1. Binding of Na^+^,K^+^-ATPase to microtiter plate coated with wheat germ agglutinin

One of earlier studies of the immobilization of Na^+^,K^+^-ATPase utilizes the high affinity binding of wheat germ agglutinin to the β-subunit of Na^+^,K^+^-ATPase [152]. By this approach, affinity of wheat germ agglutinin for binding to sugars N-linked to proteins was utilized to achieve directional binding of Na^+^,K^+^-ATPase. Wheat germ agglutinin was covalently bound to the surface of microtiter plate. After addition of Na^+^,K^+^-ATPase and binding of its β-subunit to wheat germ agglutinin, the cytoplasmatic side of an enzyme was exposed on the surface. Also, activity of Na^+^,K^+^-ATPase measured by the hydrolysis of p-nitrophenylphosphate was preserved, as well as the sensitivity to ouabain. The amount of Na^+^,K^+^-ATPase bound to the microtiter plate was higher than that used in the direct immobilizing systems. This technique might be exploited for the screening of monoclonal antibodies against cytoplasmatic domains of Na^+^,K^+^-ATPase.

### 3.2. Functional immobillization of biomembrane fragments on planar waveguids

The method for the functional immobilization of Na^+^,K^+^-ATPase-rich membrane fragments isolated from pig kidney and from the rectal gland of dogfish, on planar metal oxide waveguides was developed [153]. To our best knowledge, this is the first study on the immobilization of Na^+^,K^+^-ATPase -containing membrane discs that results with flat surface configuration and with fully preserved protein activity.

**Figure 7.**
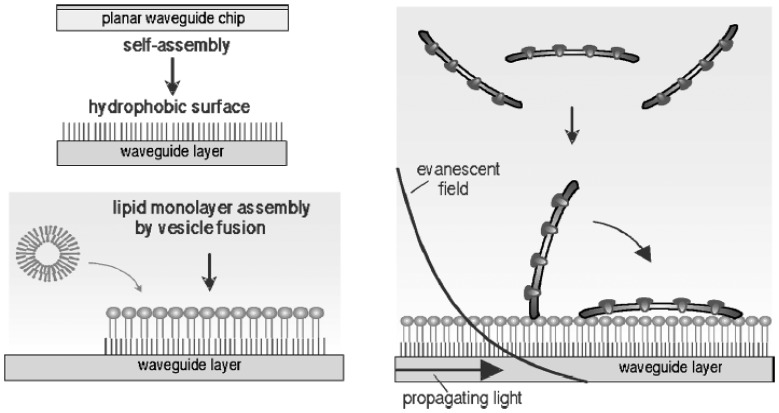
The concept of the functional immobilization of membrane fragments on the metal oxide waveguide. First step (left) formation of a biocompatible surface by self-assembly of a lipid-monolayer. Second (right) stable sorption of membrane fragments onto the preformed lipid interface. Reproduced from [153].

To summarize, the integral membrane proteins can be successfully immobilized on the flat surface without application of any detergent (for instance, sodium dodecyl sulphate, SDS) for partial solubilization of membranes. The concept of the method is schematically presented in (Figure 7). Briefly, authors have prepared a biocompatible surface of by deposition of phospholipid monolayer on the surface of metal oxide (Ta_2_O_5_) followed by the sorption of membrane fragments on this interface. By this approach, the natural milieu of Na^+^,K^+^-ATPase is preserved, which enables much more controlled conditions for the enzyme immobilization with respect to its membrane orientation.

Besides functional studies focused on the activity of immobilized Na^+^,K^+^-ATPase and its sensitivity towards ouabain, method of immobilizing Na^+^,K^+^-ATPase on the sensitized surface of metal oxide waveguide allowed the fluorescence study on the surface distribution of the enzyme and its orientation in the membrane, as well as the mapping of binding sites located on the enzyme, which in a best way resemble the binding of a ligand under natural conditions. Kinetic study of binding of K^+^-ion to the Na^+^,K^+^-ATPase revealed fully preserved enzyme activity and conformation after binding to the sensitized surface of waveguide. Although not studied so far, this method can be conveniently used for investigations of molecular aspects of interaction of various substrates and ligands with Na^+^,K^+^-ATPase, but also for other integral membrane proteins, such as membrane receptors.

In another study, uncoated surface of polymer furan was used as a support for immobilization of membrane fragments containing also Na^+^,K^+^-ATPase [154]. These membrane fragments were isolated from kidney tissue and they were immobilized on the furan surface by adsorption. However, this study was not focused on the functional immobilization of Na^+^,K^+^-ATPase, but to find conditions for scanning force microscopy (SFM) of living cells and membranes and surface with low roughness was obtained.

### 3.3. Immobilization of brain Na^+^, K^+^-ATPase on nitrocellulose, glass fiber and polyvinylidene fluoride membranes

The activity of Na^+^,K^+^-ATPase partially purified from synaptosomal plasma membranes (SPM) from rat brain and immobilized on the surface of three different solid supports was investigated in a comprehensive study of Momić *et al.* [115]. Nitrocellulose (NC), glass fiber (GF) and polyvinylidene fluoride (PVDF) membranes were used as carriers for the adsorption of the SPM Na^+^,K^+^-ATPase. The procedure for enzyme immobilization to these supports was simple; basically, the driving force is the physical adsorption either by incubation of the enzyme solution or adsorption was facilitated by the use of vacuum system. PVDF membrane has to be rinsed and activated with methanol prior to binding. Effects of the temperature and pH of the solution on the efficacy of binding were studied as well.

The Na^+^,K^+^-ATPase was partially purified by solubilization of the SPMs with SDS. The use of SDS at the optimal ratio to the protein (0.2 mg SDS/mg protein) [155] resulted in an increase of the enzyme specific activity by 39.423±0.933 %. The enzyme preparation obtained by this procedure was used in the immobilization experiments.

Enzyme stability, activity and sensitivity towards different compounds before and after immobilization were compared. The SPM immobilized on the NC membrane expressed the highest Na^+^,K^+^-ATPase activity in comparison to other solid supports tested in this study. The mechanism of protein immobilization on hydrophilic surface of nitrocellulose membrane can be ascribed to both charge-charge interactions and weak secondary forces (van der Waals, especially hydrogen interactions). On the other hand, the strong hydrophobic binding of the enzyme to the PVDF and GF membranes most likely causes the decrease of enzyme activity.

**Figure 8.**
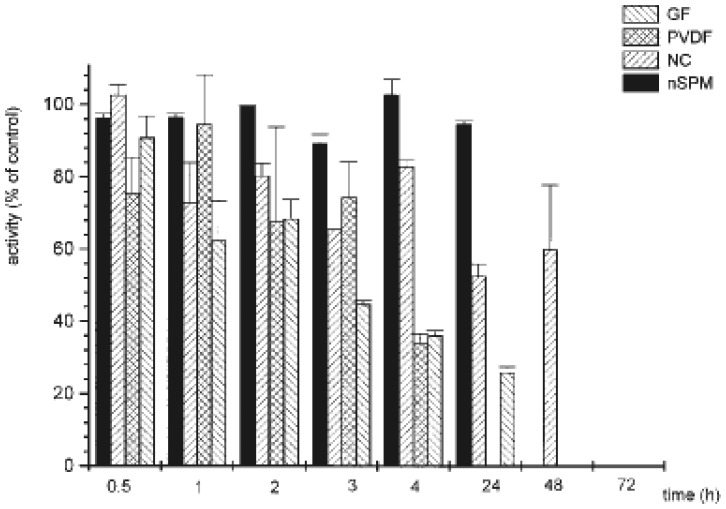
Stability of the Na^+^,K^+^-ATPase activity of native SPM (nSPM) and adsorbed SPM (aSPM) on different supports as a function of time after adsorption. Reproduced from [115].

The time-stability of Na^+^,K^+^-ATPase immobilized to the different supports was tested at 20 °C for a total period of 72 h, and the results are shown in (Figure 8). While Na^+^,K^+^-ATPase in the native preparation (non-adsorbed, nSPM) completely lost its activity after a period of 48 h, the enzyme SPM adsorbed (aSPM) on nitrocellulose showed a loss of 20% in the first 4 h and a total loss of activity within 72 h. The Na^+^,K^+^-ATPase adsorbed to the GF membrane was stable over the same period of time as the native preparation, but the percent of retained activity was much less. The activity dropped by about 30% in the first 2 h and then the loss of activity was 70 % within 24 h. The enzyme adsorbed to the PVDF membrane retained about 80 % of its activity during the first 3 h of storage, but had lost all its activity after 24 h.

Further, the effect Cd^2+^, Hg^2+^ salts and of ß-methyldigoxin on the activity of the enzyme immobilized on the NC membrane was investigated. Both metal ions and digoxin induced a concentration-dependent inhibition of the enzymatic activity. Interestingly, the enzyme immobilized on the NC membrane was found to be more sensitive to Cd^2+^ and Hg^2+^ than the native protein, whereas the sensitivity towards digoxin remained unchanged. Most likely, the conformation of enzyme has changed resulting in the increased sensitivity towards heavy metals salts. On the other hand, these conformational changes might disturb interaction of larger molecules such as digoxin with the enzyme.

The results obtained during the study imply the possibility to use the simple adsorption of Na^+^,K^+^-ATPase on the NC solid supports for potential application as biosensor. A biosensor that is based on Na^+^,K^+^-ATPase immobilized on NC support would be easy to store and use as a part of a detection kit.

### 3.4. Immobilization of Na^+^,K^+^-ATPase on the surface of polystyrene microtiter plate

Another immobilization system also developed in our laboratory is the immobilization of SPMs on the polystyrene microtiter plates [104]. The principle of this immobilization is also simple physical adsorption, *i.e.* incubation of isolated SPMs from rat brain for 18h at 4 ° C with a polystyrene matrix -microtiter plates. Results demonstrated that about 10% of the protein fraction was bound to the surface of microtiter plate, whereas Na^+^,K^+^-ATPase and Mg-ATPase retained up to 80% of their activity after immobilization. Also, the sensitivity of both enzymes towards heavy metal salts was not altered after immobilization. The only exception was the sensitivity of Mg-ATPase for the presence of Cd^2+^, which was increased in comparison to the native enzyme.

**Figure 9.**
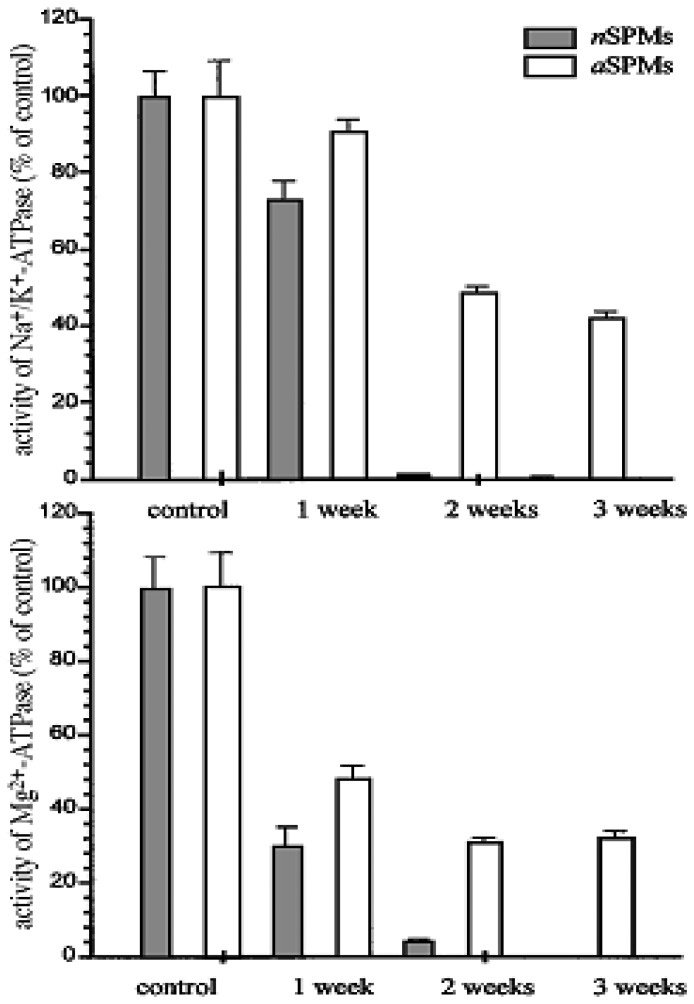
Stability of the Na^+^,K^+^-ATPase and Mg^2+^-ATPase activity of nSPM and aSPM as a function of time after the adsorption (weeks). Reproduced from [104].

Astonishingly, the stability of Na^+^,K^+^-ATPase bound to the polystyrene microtiter plate was increased compared with native SPM preparations, but also in comparison to the enzyme immobilized to the NC membranes [115]. Whereas the stability of partially purified Na^+^,K^+^-ATPase from the SPMs immobilized on the NC membrane was measured in days, the stability of Na^+^,K^+^-ATPase and Mg^2+^-ATPase in the SPMs bound to the microtiter plate could be monitored for weeks (Figure 9).

With respect to the design of a solid support, immobilization of Na^+^,K^+^-ATPase on the microtiter plate has an advantage over other supports, since it enables carrying out multiple tests simultaneously and in rather small volumes. Moreover, simplicity of the immobilization procedure resulting in high binding efficiency and long enzyme stability is an additional advantage over other methods that require pre-treatment of a surface prior to the enzyme immobilization.

Taken together, the physical adsorption of Na^+^,K^+^-ATPase either on the NC membrane or on the surface of polystirene microtiter plate might be used as practical and comfortable systems for *in vitro* toxicological investigations.

### 3.5. Immobilization of Na^+^, K^+^-ATPase on dextran surface

Another example of the Na^+^,K^+^-ATPase immobilization is the covalent binding of the enzyme to the surface of dextran [156]. Prior to addition of the Na^+^,K^+^-ATPase from dog kidney or porcine cerebral cortex, surface of dextran was activated by ethylenediaminecarbodiimide/*N*-hydroxysuccinimide (EDC/NHS) reagent. After activation of dextran surface, Na^+^,K^+^-ATPase was added and directly bound to the surface of dextran.

Although the loss of an enzyme activity occurs frequently after enzyme cross-linking (*i.e.* establishment of new covalent bonds), the activity of Na^+^,K^+^-ATPase was preserved after immobilization on dextran surface and binding of ouabain for Na^+^,K^+^-ATPase was possible. On the other hand, the binding of phycotoxin palytoxin on the Na^+^,K^+^-ATPase was most probably disrupted after this method for Na^+^,K^+^-ATPase immobilization. Taken together, adsorption of Na^+^,K^+^-ATPase -rich membrane fragments on different supports mostly resulted in the more stable enzyme which retained its activity. This indicates that binding sites for substrates, as well as its active site were preserved after enzyme immobilization. So far, minor drawback of immobilization of Na^+^,K^+^-ATPase is that the sensitivity of enzyme towards certain ligands has significantly changed, what is difference with respect to the physiological situation. In the case of palytoxin, Na^+^,K^+^-ATPase was found insensitive after immobilization on the surface of dextran. Significantly, immobilization of Na^+^,K^+^-ATPase enabled more detailed studies of enzyme function, kinetic properties of enzyme, electric properties of the membrane, orientation of enzyme in the membrane, binding of antibodies and development of new antibodies, as well as numerous toxicological studies.

## 4. Signal detection

In the next charpter, the most commonly-applied methods and comertially available kits applied for the determination of native enzyme activity were overwieved and the signal detection Na^+^,K^+^-ATPase based biosensors for potential medical and pharmaceutical applications are presented. Besides, some methods that can be also applied for study of the interaction between various ligands and Na^+^,K^+^-ATPase are shortly presented.

### 4.1. Methods for determination of Na^+^,K^+^-ATPase activity

#### 4.1.1. Spectroscopic methods

Most of the colorimetric methods are based on the spectrophotometric determination of free orthophosphate (Pi) released due to the Na^+^,K^+^-ATPase catalyzed hydrolysis of ATP. Pi is usually transformed to a phosphomolybdate complex in an acid medium followed by a reduction with ascorbic acid or stannous chloride followed by complexation with basic dyes that yield colored complexes [60-62]. As such, only static end-point determinations can be performed by measuring released inorganic phosphate generated during ATP hydrolysis by ATPases during the incubation period [157].

The simple, sensitive and reproducible [158] method based on the reduction of a phosphomolybdate complex by Elon in a copper acetate buffer avoids the interference by ATP with color development. There is also less or no interference by other compounds usually present in ATPase assay media. Moreover, the determination of association, dissociation and affinity constants are much easier and faster compared with ELISA and RIA [63].

The electron spin resonance (ESR) spectra of spin-labeled lipid molecules provide a method of probing the molecular interactions at the protein-lipid interface in biological membranes. This method enables to obtain structural information upon evaluation of dipolar interaction of bound radicals as well as information about nucleotide binding characteristics of the proteins, such as substrate binding stoichiometries and affinities. Lipid spin labels have been used to study lipid-protein interactions in bovine and frog rod outer segment disc membranes, in Na^+^,K^+^-ATPase membranes from shark rectal gland, and in yeast cytochrome oxidasedimyristoyl phosphatidylcholine complexes [159]. These systems all display a two component ESR spectrum from 14-doxyl lipid spin-labels. One component corresponds to the normal fluid bilayer lipids. The second component has a greater degree of motional restriction and arises from lipids interacting with the protein. For the phosphatidylcholine spin label there are effectively 55 ± 5 lipids/200,000-dalton cytochrome oxidase, 58 ± 4 mol lipid/265,000 Dalton Na^+^,K^+^-ATPase, and 24 ± 3 and 22 ± 2 mol lipid/37,000 dalton rhodopsin for the bovine and frog preparations, respectively. These values correlate roughly with the intramembrane protein perimeter and scale with the square root of the molecular weight of the protein. Experiments with spin labels of different head groups indicate a marked selectivity of Na^+^,K^+^-ATPase for stearic acid and for cardiolipin, relative to phosphatidylcholine.

Reaction-induced infrared (IR) difference spectroscopy with caged ATP and Na^+^,K^+^-ATPase was used to differentiate between phosphorylated and unphosphorylated states of the enzyme as well as of its ouabain complex [62]. The IR spectral changes upon phosphoenzyme formation were characterized and interpreted. The results showed that high Na^+^ concentrations prevented the binding of ouabain with high affnity, which is consistent with the results of a corresponding kinetic study employing spectrofluorimetry and calorimetric titrations. Changes of protein structure and protein microenvironment associated with partial reactions of Na^+^,K^+^-ATPase were investigated employing reaction-induced Fourier transform infrared (FTIR) difference spectroscopy in aqueous solution between 2,000 and 950 cm^-1^. The reaction was initiated by the ultraviolet (UV)-photochemical release of ATP from a precursor such as caged ATP. Employing this method, protonation and phosphorylation reactions can be detected in a convenient way without using any labeling methods.

#### 4.1.2. HPLC methods

The high performance liquid chromatography (HPLC) assays for determination of ATPase activity were developed and validated [160, 161]. The method is based on the measuring of the products of ATP hydrolysis (mainly ADP), released during the reaction, by HPLC. This method presents the important advantages for the assay of the enzymes since it is reproducible through time, perfectly linear, and is extremely sensitive. The substrate, ATP, was incubated with the enzyme preparation in the presence of an inhibitor. After stopping the enzyme reaction, the incubation mixture was filtered through a membrane filter, and ADP and ATP in the filtrate were separated by HPLC. The inhibitory effect on the enzymatic reaction was estimated by measuring the peak area ratio of ADP to ATP on the chromatogram [160].

The proposed assay method has proved to be satisfactory with respect to simplicity, sensitivity, and reproducibility. In addition, when ouabain was used as an inhibitor, the HPLC method was found to be applicable for Na^+^,K^+^-ATPase activity determination. This indicated that this HPLC assay would enable determination of ATPases other than Na^+^,K^+^-ATPase, when other inhibitors are employed instead of ouabain.

The HPLC procedure was sensitive, precise and linear calibration graph was obtained over the range of protein amount of the enzyme source studied, the intra- and inter-assay variations being lower than 10%. Also, even when the samples were contaminated with Pi, the HPLC method allowed determination of ATPase activity. This method is also very versatile, since it allowed to assess the Km value for Ca^2+^ of the Ca^2+^,Mg^2+^-ATPase in some tissues [161].

**Figure 10.**
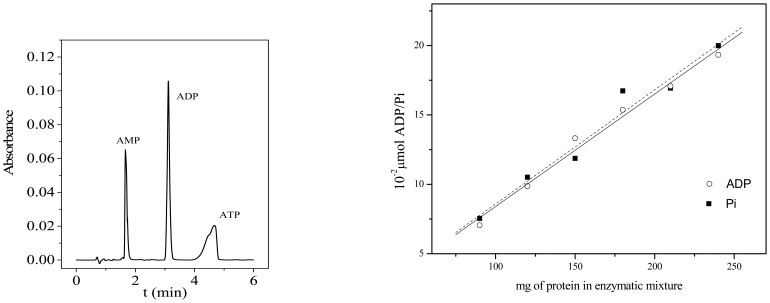
Chromatogram of 5 mM ATP hydrolysis products by using UPLC and comparison of UPLC and spectrophotometric determination of products of ATP hydrolysis catalyzed by Na^+^,K^+^-ATPase. Reproduced from [162].

Besides, an ultra performance liquid chromatograpfy (UPLC) method for determination of ATP, AMP and ADP in Na^+^,K^+^-ATPase assay was developed [162]. Parallel, inorganic phosphorus (Pi) released from ATP was spectrophotometrically determined (Figure 10). The values that were obtained by the two methods revealed a significant correlation. In addition, when ouabain was used as an inhibitor, the HPLC method was found to be applicable for Na^+^,K^+^-ATPase activity determination [160].

#### 4.1.3. Coupled enzymatic methods

There are a large number of assays in cellular and molecular biology based on measurements of either produced or consumed inorganic phosphate [59]. Therefore, the ability to quantitatively measure these phosphorous compounds can allow the assessment of the associated enzymes. ATPase assays can be performed by coupled spectrophotometric assay for adenosinediphosphate (ADP) with pyruvate kinase, lactate dehydrogenase and luciferase.

**Figure 11.**
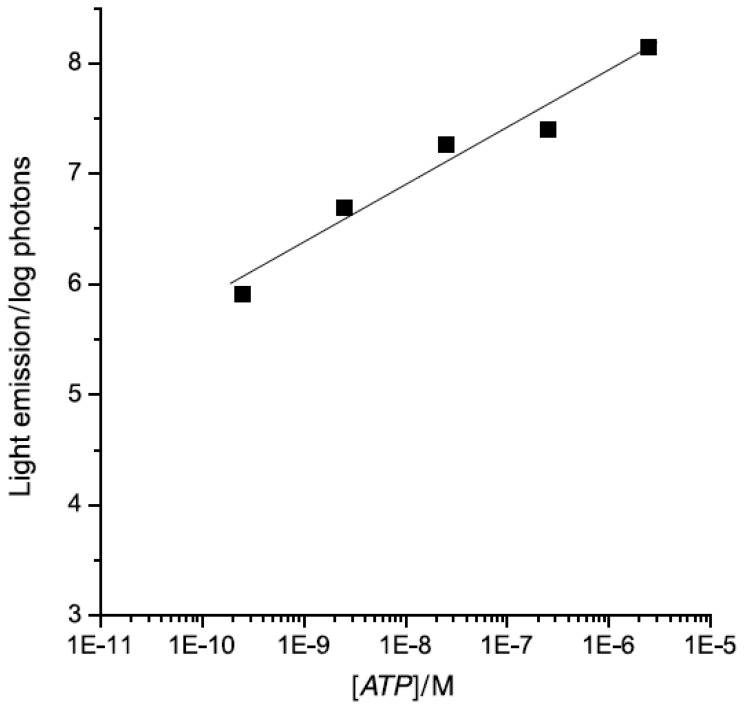
Representative ATP standard curve for light emission as function of ATP concentration in the range of 1×10^-10^ to 1×10^-5^ M. Reproduced from [163].

A bioluminescence assay was applied for fast and sensitive evaluation of heavy and transitional metals (Cu^2+^, Pb^2+^, Cd^2+^, Hg^2+^) effect on the rat brain synaptosomal membrane Na^+^,K^+^-ATPase activity [163]. The assay consists of ATP-consuming reaction catalyzed by synaptic plasma membrane Na^+^,K^+^-ATPase coupled to the luminescent firefly luciferase, which consumes residual ATP after the course of Na^+^,K^+^-ATPase reaction. The bioluminescence Na^+^,K^+^-ATPase assay was applied to study the effect of on rat brain Na^+^,K^+^-ATPase activity after assay optimization (Figure 11). The fast bioluminescence Na^+^,K^+^-ATPase assay with small sample and substrate requirements could be adjusted for high-throughput environmental and pharmacological screening.

The method modified to be performed in microplates [164] is based on coupled enzymatic reaction with the enzyme, purine-nucleoside (orthophosphate ribosyltransferase, EC 2.4.2.1 (PNP)). This enzyme uses inorganic phosphate (Pi) to convert the substrate 2-amino-6-mercapto-7-methylpurine riboside (MESG) to ribose 1-phosphate and 2-amino-6-mercapto-7-methylpurine. The enzymatic removal of the ribose moiety from MESG results in a shift in the wavelength of maximum absorbance from 330 nm to 360 nm [64, 165]. Assuming there is no preexisting phosphate, any increase in the absorbance at 360 nm must be the result of Pi liberation from ATP hydrolysis.

Assays were initiated by the addition of reaction substrate and immediately placed in a μQuant Scanning Microplate Spectrophotometer. Data capture and reader control were carried out using KC4 software (BioTek Instruments). Contamination of reagents with small amounts of Pi can be accounted for by subtraction of a reagent blank, if the standard curve and samples all contain equivalent amounts.

However, the use of UV transparent microplates and the subtraction of a reagent blank can virtually eliminate these problems. Unlike most assays that quantitate inorganic phosphate, this assay is performed under physiologic conditions. This allows for real-time kinetic analysis of ATP-dependent enzymatic reactions.

#### 4.1.4. Fluorescence and bioluminescence methods

A number of physical sensing techniques, preferentially based on fluorescence and bioluminscence detection and designed for high throughput screening even of unpurified synaptosomes isolated from weighed microsamples (2–3 mg wet weight of tissue), have been developed [164, 166]. Therefore, and due to the trend of integrating a larger and larger number of assays on a single detection plate [167] leading to increase surface/volume ratios in an assay well, surface-sensitive fluorescence techniques [168-170] become increasingly important.

The method is based on fluorometric measurement of ADP formed in the course of the Na^+^,K^+^-ATPase reaction. It is highly sensitive and can be used to determine the Na^+^,K^+^-ATPase activity of membrane preparations with a protein content of 0.5–1.0 μg per sample [170].

A method to measure high-affinity binding sites for fluorescent ligands was [171] was applied to the determination of Na^+^,K^+^-ATPase using the fluorescent ouabain derivative anthroylouabain. The method consists of measurements of the fluorescence intensities of a saturating concentration of anthroylouabain in the presence and absence of Na^+^,K^+^-ATPase and ouabain. These data and the fluorescence enhancement factor upon anthroylouabain binding are used to calculate the concentration of binding sites. The measurements can be done in a few minutes and 10 to 100 p moles of ouabain sites is sufficient for accurate determinations. Because phosphorylation of Na^+^,K^+^-ATPase is necessary to bind anthroylouabain, only functional enzyme was detected by this method [172].

The fluorescence detection was applied on Na^+^,K^+^-ATPase isolated as the major protein in nanoparticulate membrane fragments (discs of ca. 250 nm mean diameter) from specialized tissue such as kidney or salt glands. In contrast to solubilized systems, the protein is in its native membrane environment and retains its original biomembrane orientation [153]. The isolated membrane discs, however, are surrounded by the same aqueous medium on both sides and are no longer capable of separating the two different aqueous cell compartments. A fluorescent marker, such as fluorescein-5-isothiocyanate (FITC) specifically binds to a single lysine residue, located within the ATP binding domain, for monitoring binding events by fluorescence quenching [173, 167, 174]. The fluorescence of the labeled Na^+^,K^+^-ATPase changes characteristically upon binding of different ligands and cations and was also used here for the analytical detection.

### 4.2. Na^+^, K^+^-ATPase based biosensors

#### 4.2.1. Detection of various analytes by Na^+^,K^+^-ATPase assay

Na^+^,K^+^-ATPase is sensitive to a great number of different groups of analytes, including cardiovascular drugs, biologically important elements, heavy metals, organic solvents and some toxic organic compounds [102]. The possibility of using ATPase system as a biological component for multi-response sensing system for detection of different compounds is based on the level of change of enzyme activity in the presence of analytes. The series of ATPase assays containing the components as given in Table 3 were prepared in 96 wells microtiter plate in order to reconsider the response of the enzymes in the presence of the investigated compounds by single exposure. The absorbance was measured and compared to the control value [102].

The method was applied to the determination of Na^+^, K^+^, Mg^2+^, and heavy metals (as the group of elements) in mineral water. The cuvette test was applied, and four reaction mixtures were prepared containing the sample. Each mixture contained the standard concentrations of medium assay components, excluding the target ion (Na^+^, K^+^, and Mg^2+^) for alkaline elements detection. After the colorimetric assay was added, the absorbance at 690 nm was measured and compared to the absorbance of the control solution Table 4). The proposed method was also applied to the quality control of digoxin. The tested sample of Lanoxin injection was added to the standard medium assay containing 1mM EDTA. The activity was measured as described, and compared to the control value. The results presented in Table 3 showed the same result as obtained by standard method (HPLC) with the mean standard deviation of 6.45%.

However, the cuvette test for detection of analytes is simple and potentially useful for quick measurements outdoors, too. Consequently, the test may be put under the consideration for the quality control by cardiotonic drugs production, as well as for detection of ATP, K^+^, Mg^2+^ and Na^+^ ions in biological fluids.

A simple Na^+^,K^+^-ATPase assay is described as a suitable method for testing of digoxin photodegradation [175]. The exposure of Na^+^,K^+^-ATPase to the photodegraded samples exhibited reduced inhibition of the enzyme, compared to the non irradiated samples containing equal initial concentrations of drug. The degree of inhibition was dependent on the irradiation time. The concentrations of digoxin in irradiated samples were evaluated by HPLC analysis. Excellent agreement of the results obtained by both methods was observed. The investigation of the influence of irradiated samples on Na^+^,K^+^-ATPase inhibition revealed no side products acting as Na^+^,K^+^-ATPase inhibitors.

#### 4.2.2. Resonant mirror biosensor

The resonant mirror biosensor was used to evaluate the binding of ouabain and palytoxin to immobilized Na^+^,K^+^-ATPase [125]. Na^+^,K^+^-ATPase from dog kidney or porcine cerebral cortex, was immobilized directly to a carboxymethyl dextran (CMD) cuvettes [156,176]. Association measurements were made by using an Iasys Affinity Sensor (Labsystem, UK), following the procedures recommended by the manufacturer. The instrument analyzes interactions occurring within a few hundred nanometers from the sensor surface using evanescent waves, to measure changes in refractive index at the sensor surface. These changes can be measured as shifts in the resonance angle, expressed as arc seconds. Na^+^,K^+^-ATPase was coupled to CMD cuvettes, in a total volume of 60 μL.

The optical biosensor has dual cuvette surfaces that allow simultaneous dual-channel monitoring. By subtracting the sensor response of the control channel, the responses caused by nonspecific binding and bulk refractive index changes could be corrected. The stirred cuvette system used in this instrument ensures that mass transport effects during binding are minimized. The data-sampling interval was 0.3 s and the stirring rate was 85 rpm controlled by the Iasys software.

**Figure 12.**
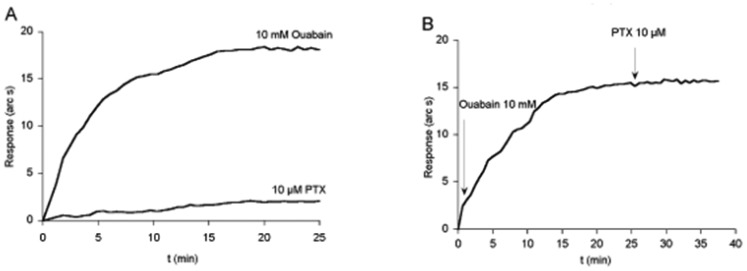
Porcine cerebral cortex Na^+^,K^+^-ATPase -ouabain/PTX association. (A) Association curves of 10mM ouabain or 10 μM palytoxin added over immobilized Na^+^,K^+^-ATPase. (B) Association curve after addition of 10mM ouabain (first arrow) over immobilized Na^+^,K^+^-ATPase. PTX of 10 mM was added 25 min later (second arrow). Reproduced from [156].

A range of PTX or ouabain concentrations, were allowed to bind with the immobilized Na^+^,K^+^-ATPase, and the association curves were monitored for 25 min (Figure 12). Subsequently, the cuvette was washed with PBS and the dissociation phase was monitored for 5 min. The cuvette was finally regenerated by washing in the presence of 0.01 M HCl. Since the instrument has a dual optical path, all experiments were performed in one cuvette and, simultaneously, the second cuvette was used as control to normalize data by subtracting the response of this control cuvette with medium from the response of the cuvette with toxin. When the Na^+^,K^+^-ATPase from porcine cerebral cortex was immobilized, the same results were obtained in the presence of different concentrations of either ouabain or PTX (Figure 12). Ouabain induced an association curve that reached a signal of 18.1 arcsec, while PTX did not induce any change in the association curve. This clearly indicates that PTX does not modify the binding of ouabain.

#### 4.2.3. Evanescent wave fluorescence biosensor

Evanescent wave sensors using planar waveguides collect fluorescence emission perpendicular to the waveguide whereas most fiber optic biosensors of this type collect the emitted light out the end of the fiber [153,177]. The optical technique based on the highly sensitive detection of surface-confined fluorescence in the evanescent field of the waveguide was used to investigate the interactions of the Na^+^,K^+^-ATPase rich membrane fragments immobilized on planar metal oxide, with cations and ligands. The combination with surface-confined fluorescence detection by planar waveguides allowed the simultaneous investigation of side-directed ligand binding and of functional properties of Na^+^,K^+^-ATPase. After successful immobilization of the membrane fragments, the functional activity of the immobilized protein was probed by monitoring the fluorescence changes upon specific, side-directed binding of cations and ligands using the FITC-labeled ATPase. The enzyme functional activity was tested by selective K^+^ cation binding, interaction with anti-fluoerescein antibody 4-4-20, phosphorylation of the protein and binding of inhibitory ligand ouabain [153]. The described approach offers great advantages because no detergent solubilization of the protein is necessary, as is the case for other reconstitution procedures, because the assays can be optimized efficiently and because fast and reproducible measurements can be performed, due to the easy exchange of the aqueous media for the different assays. The orientation of the immobilized membrane fragments on the surface was finally concluded from the comparison of the fluorescence changes, measured with the PWG sensor, with those found with the same membrane sample in bulk solution.

Fluorophore labels of the surface-bound enzyme can be selectively excited in the evanescent field. For example, a fluorescent marker, such as fuorescein-5-isothiocyanate (FITC) can be specifically bound to a single lysine residue, located within the ATP binding domain, for monitoring binding events by fluorescence quenching [178-180]. The fluorescence of the labeled Na^+^,K^+^-ATPase changes characteristically upon binding of different ligands and cations and was also used for the analytical detection.

#### 4.2.4. Optical voltage assay of Na^+^,K^+^-ATPase

Na^+^,K^+^-ATPase generates the Na^+^ and K^+^ gradients across the plasma membrane of cells, which are needed to establish negative resting membrane potentials. In an effort to develop a high-throughput cell-based functional assay for the Na^+^,K^+^-ATPase, optical techniques was used to develop a membrane potential assay of pump activity [181]. Wild-type CHO-K1 cells were stained with FRET-based voltage-sensitive dyes and pump dependent changes in membrane potential were assayed on a VIPRTM fluorescent plate reader. Cells were incubated to block the Na^+^/K^+^ pump and to allow for intracellular Na^+^ accumulation. Addition of a K^+^-containing extracellular solution reactivated the Na^+^/K^+^ pump and produced a fluorescence resonance energy transfer (FRET) increase indicating cell hyper polarization. Ouabain completely blocked this effect. Electrophysiology confirmed the optical findings as recordings current of 175-300 pA capable of hyperpolarizing the CHOK1 cell.

An innovative method for identifying a Na^+^ channel blocker generally includes providing a cell containing a Na^+^ channel [182, 183]. The channel demonstrates both transient and persistent currents. The cell also includes a potassium K^+^ channel and a Na^+^,K^+^-ATPase (Na^+^ pump). A fluorescent dye is disposed into the well. The fluorescent dye is sensitive to change in cell membrane potential in order to enable optical measurement of cell membrane potential. A Na^+^ channel blocker, to be assayed, screened or otherwise identified, is added to the well and stimulation current is passed through the cell in an amount sufficient to generate an action potential before and after the addition of the Na^+^ channel blocker. Thereafter, a change in cell membrane potential is optically measured.

Apparatus in accordance with the invention [183] includes a screen for identifying a Na^+^ channel blocker. The screen includes at least one cell comprising a Na^+^ channel, the channel demonstrating both transient and a persistent current. In addition, the cell further comprises a potassium (K^+^) channel and a Na^+^,K^+^-ATPase (Na^+^) pump. At least one well containing the cell is provided. A fluorescent dye sensitive to change in cell membrane potential in order to enable optical measurement of cell membrane potential is also included. Electrodes disposed in the well are in the well are provided for passing a stimulating current through said cell sufficient to generate an action potential before and after the addition of the Na^+^ channel blocker, to be identified, to said cell.

These assays can be performed using robotic systems that are frequently used for high throughput screens in the pharmaceutical industry. The chances for discovering novel compounds that block or modify persistent Na^+^ currents while sparing transient Na^+^ currents should be measurably increased. Compounds that are selected by the above screens may then be examined in great detail using conventional electrophysiological methods for further examination and ultimate selection of a lead structure.

#### 4.2.5. High-throughput screening assay for Na^+^, K^+^-ATPase using atomic absorption spectroscopy

An innovative method of chemical analysis involving Flame Atomic Absorption Spectroscopy (FAAS) and Graphite Furnace Atomic Absorption Spectroscopy (GFAAS) in combination with flux assays to directly measure intracellular or extracellular ion concentration to analyze Na^+^,K^+^-ATPase activity [184] was developed. The invention pertains to experimental methodologies for biopharmaceutical research, particularly for the analysis of drug candidates for therapeutic effects on Na^+^,K^+^-ATPase. The invention describes a method of preparing sample cell cultures for analysis, and using a unique flux (intake of ions) assay and the techniques of flame atomic absorption spectrometry (FAAS) or graphite furnace atomic absorption spectrometry (GFAAS) to directly measure the intracellular ion content of those cell cultures, enabling the measurement of ion flux and ion channel activity. The method enables the measurement of ion flux through cell membrane Na^+^,K^+^-ATPase to provide information on Na^+^,K^+^-ATPase activity.

The first method of preparing the cell culture samples for analysis is called the Tracer Ion Method. The rubidium ion and lithium ions are similar to the potassium ion and sodium ion, respectively, both in terms of physical and chemical properties such as molecular size and ionic charge. In view of the tracer ions, sodium or potassium may be called native ions. Because of these similarities, these ions are able to pass through many respective channels and transporters, with varying permeability coefficients. Measurement of the concentration of the tracer ion provides a measure of Na^+^,K^+^-ATPase activity.

The second method for preparing cell culture samples for analysis is called the Direct Measure Method. The cell cultures are incubated with potassium ions instead of tracer ions. Therefore, when the ion concentration is eventually measured, it will be a direct measurement of potassium ion movement into the cell through the Na^+^,K^+^-ATPase activity.

An advantage of this invention is that the experimental methodology provides a high throughput way for scientists to accurately determine the therapeutic effects of novel candidate compounds for screening against Na^+^,K^+^-ATPase target.

## Conclusions

Na^+^,K^+^-ATPase is the protein specially abundant in nerve cells, and represents the excellent model system for elucidation of neurotoxic effects, as well as the antitumor potency of the various modulators of its activity. The great number of organic compounds and inorganic salts, including cardiovascular and anti-cancer drugs, biologically important elements, heavy metals, organic solvents and some toxic organic compounds, such as pesticides and herbicides, strongly modulate enzyme activity on the concentration dependent manner. Because of its high sensitivity to the broad spectrum of toxic compound, as well as potential cardiotonic and anticancer drugs, Na^+^,K^+^-ATPase activity can be taken as meaningful index of cellular activity and forms a useful toxicological tool in medicine, pharmacy and environment.

In order to fully utilize the sensitivity of this enzyme towards a broad spectrum of compounds, its properties were improved by immobilization. The immobilization approaches improved rather low time and temperature stability of Na^+^,K^+^-ATPase, and also preserved the enzyme properties such as structure, enzyme activity and selectivity, particularly of the enzyme present in the synaptosomal plasma membranes. Procedures for immobilization of Na^+^,K^+^-ATPase are mostly based on the simple physical adsorption or the covalent cross-linking of membrane fragments to the surface of support, such as planar waveguids, wheat germ agglutinin, nitrocellulose, glass fiber, polyvinylidene fluoride membranes, polystyrene microtiter plate and dextran surface.

In this review, we have given a literature survey on: *i)* the functional properties of Na^+^,K^+^-ATPase, *ii)* the inhibitory potential of a spectrum of compounds and their mechanism of action, *iii)* the different immobilization procedures applied particularly for this enzyme, as well as *iv)* the methods commonly applied for determination of the Na^+^,K^+^-ATPase activity and for monitoring of the structural changes of the enzyme upon interaction with various substances. Taken together, one can conclude that the most important pre-requisites to apply Na^+^,K^+^-ATPase as a biological tool for applications in medical, pharmaceutical and environmental research already exist. First of all, the enzyme demonstrated very high sensitivity towards a spectrum of compounds, secondly, immobilization procedures appeared to be rather efficient and yield the active and stable enzyme and, finally, methods for measurement of the Na^+^,K^+^-ATPase activity are developed and in common use. In addition, some of the methods are very convenient for outdoor applications. On the other hand, lack of selectivity for various toxic compounds makes potential applications of Na^+^,K^+^-ATPase for enzyme-based biosensor rather difficult and implies its application only as a model system for sensing toxic compounds and for elucidating potential toxic effects. Although well known and broadly studied, Na^+^,K^+^-ATPase will hardly be seriously considered for development of a future biosensor.

## Figures and Tables

**Table 1.** The IC_50_ for the representative of some characteristic groups of sodium pump inhibitors.

Inhibitor	IC_50_ (M)	Tissue	Method	Ref.

***Cardiotonic glycosides***				

Ouabain	1.5×10^-9^	cardiac tissue	coupled enzyme assay	[51]
	9.4×10^-6^	CHO-K1 cells	FRET	[181]

digoxin	a2.5×10^-8^	rat brain SPM	coupled enzyme assay	[49]
	b1.3×10^-4^			
	a4.6×10^-6^	human erythrocytes	spectrophotometric	[67]
	b1.0×10^-3^			

Gitoxin	a2.8×10^-7^	human erythrocytes	spectrophotometric	[67]
	b4.1×10^-4^			

metildigoxin	2.9×10^-3^	rat brain SPM	spectrophotometric	[78]

***Pharmaceuticals***				

propranolol	3.7×10^-3^	rat brain SPM	spectrophotometric	[78]
verapamil	1.9×10^-3^	rat brain SPM	spectrophotometric	[78]
oleandrin		rat brain SPM	spectrophotometric	[78]
promethazine	8.4×10^-4^	rat brain SPM	spectrophotometric	[78]

		***Metal ions***		

Cu^2+^	5.9×10^-6^	rat brain SPM	bioluminiscence	[163]
Hg^2+^	1.1×10^-6^	rat brain SPM	immobilized enzyme	[104]
Cd^2+^	2.0×10^-6^	rat brain SPM	coupled enzyme assay	[118]
Pb^2+^	7.0×10^-6^	red cell membranes	spectrophotometric	[118]

***Platinum anticancer drugs***				

[PdCl(dien)]+	1.2×10^-4^	Porcine cerebral cortex	spectrophotometric	[101]
[PdCl_4_]^-^	2.3×10^-5^	Porcine cerebral cotrex	spectrophotometric	[101]
[AuCl_4_]^-^	6.9×10^-5^	dog brain microsomes	spectrophotometric	[68]
	5.1×10^-5^	human kidney homogenate		
cis-platinum	7.0×10^-4^	Ca9-22 cells	spectrophotometric	[106]

***Pesticides***				

2,4-dichlorophenoxy-acetic acid	<4.0×10^-3^	Erythrocyte	spectrophotometric	[54]
2,4,5-trichlorophenoxy-acetic acid	<4.0×10^-3^	Erythrocyte	spectrophotometric	[54]
parathion	a71.0×10^-6^	pig kidney	spectrophotometric	[142]
	b85.0×10^-6^			

a high affinity;

b low affinity

**Table 2.** IC_50_ values (μM) for Na^+^,K^+^-ATPase inhibition by some metal ions and free activation energy (∆G^°′^) (kJ mol^-1^) for water exchange and solubility constants (K_so_) metal sulphides.

Metal ion	IC_50_ (μM)^[103^]		Free activation energy (∆G°^'^)(kJ mol^-1^)^[116]^	Solubility constant (log Kso)^[116]^

	Exp.	Calc.		
Fe^2+^	34	-	38.9	-18.7
Co^2+^	168	75	40.6	-17.5
Cu^2+^	7.1	0.6	/	-33.9
Zn^2+^	22	13	29.3	-24.4
Hg^2+^	0.7		/	-48.8
Cd^2+^	1		/	-27.3
Pb^2+^	15		/	-26.7
Mg^2+^	/		44.3	/
Na^+^	/		16.7	/
K^+^	/		14.2	/

**Table 3.** Spectrophotometric response of the colorimetric assay in the presence of single analytes as the function of composition of ATPases assay. Reproduced from [102].

analyte	conc./*M*	composition of *ATP*ase assay^a^	target enzyme	relative absorbance (% of control)	

				Found	Expect.^b^
Na^+^	1×10^−2^	K, Mg, *EDTA*, *ATP*	Na^+^/K^+^-*ATP*ase	50 ±3	47
K^+^	1×10^−3^	Na, Mg, *EDTA*, *ATP*	Na^+^/K^+^-*ATP*ase	68 ±2	65
Mg^2+^	1×10^−4^	*ATP*, *EDTA*	Mg^2+^-*ATP*ase	49 ± 3>	52
Heavy metals^c^	1×10^−5^	Na, K, Mg, *ATP*	Na^+^/K^+^-*ATP*ase	0±1	0
		Na, K, Mg, *ATP*, *EDTA*	Na^+^/K^+^-*ATP*ase	100 ±1	100
pyridine	1×10^−3^	Na, K, Mg, *ATP*, *EDTA*	Na^+^/K^+^-*ATP*ase	130 ±5	130
		Mg, *ATP*, *EDTA*	Mg^2+^-*ATP*ase	120 ±4	120
urea	1×10^−3^	Na, K, *Mg*, *ATP*, *EDTA*	Mg^2+^-*ATP*ase	130 ± 5	130
		Mg, *ATP*, *EDTA*	Mg^2+^-*ATP*ase	100	100
chlorpyrifos	1×10^−5^	Mg, *ATP*, *EDTA*	Mg^2+^-*ATP*ase	52 ±3	50
digoxin	1×10^−6^	Na, K, Mg, *ATP*, *EDTA*	Mg^2+^/K^+^-*ATP*asee	90 ±2	90
		Mg, *ATP*, *EDTA*	Mg^2+^-*ATP*ase	100	100
gitoxin	1×10^−6^	Na, K, Mg, *ATP*	Na^+^/K^+^-*ATP*ase	35 ±3	35
		Mg, *ATP*, *EDTA*	Mg^2+^-*ATP*ase	100	100

a Standard concentrations for ATPase assay;

b values from [102];

c summary concentration of ions.

**Table 4.** Application of ATPases assay to real samples. Reproduced from [102].

target analyte	sample	labelled content/*M*^a^	found content/*M*^b^
Na^+^	mineral water	1.96× 10^−2^	(1 5±0.5)× 10^−2^
K^+^	mineral water	1.64×10^−3^	(2.2±0.4)× l0^−3^
Mg^2+^	mineral water	2.58×l0^−3^	(2.4±0.4)× l0^−3^
heavy metals	mineral water	-	not found
digoxin	Lanoxin^R^ injection	0 32×10^−3^	(0.31 ± 0.02) ×10^−3^

a Contents were checked by standard methods;

7 mean of 3 replicates.
