# Vasić, V. *et al.* Na^+^,K^+^-ATPase as the Target Enzyme for Organic and Inorganic Compounds. *Sensors* 2008, 8, 8321-8360

**DOI:** 10.3390/s90100377

**Published:** 2008-01-08

**Authors:** Vesna Vasić, Tatjana Momić, Marijana Petković, Danijela Krstić

**Affiliations:** 1 Vinča Institute of Nuclear Sciences, Department of Physical Chemistry, 11001 Belgrade, Republic of Serbia; E-Mails: momict@vinca.rs. (T. M.); marijanapetkovic@vinca.rs (M. P.); 2 Institute of Medicinal Chemistry, University School of Medicine, University of Belgrade, Višegradska 12, Belgrade, Republic of Serbia; E-Mail: krsticdana@yahoo.com (D. K.)

We found that reference 94 was incorrectly cited in our paper published in *Sensors* recently [1]. Therefore, reference 94 is corrected as follows:

94. Van Quaquebeke, E.; Simon, G.; Andr, A.; Dewelle, J.; El Yazidi, M.; Bruyneel, F.; Tuti, J.; Nacoulma, O.; Guissou, P.; Decaestecker, C.; Braekman, J.C.; Kiss, R.; Darro, F. Id entification of a novel cardenolide (2″-oxovoruscharin) from Calotropis procera and the hemisynthesis of novel derivatives displaying potent in vitro antitumor activities and high in vivo tolerance: structure-activity relationship analyses. *J. Med. Chem.* **2005**, *48*, 849-56.
